# Dynamic metabolic profiling of the marine microalga *Chlamydomonas* sp. JSC4 and enhancing its oil production by optimizing light intensity

**DOI:** 10.1186/s13068-015-0226-y

**Published:** 2015-03-18

**Authors:** Shih-Hsin Ho, Akihito Nakanishi, Xiaoting Ye, Jo-Shu Chang, Chun-Yen Chen, Tomohisa Hasunuma, Akihiko Kondo

**Affiliations:** Organization of Advanced Science and Technology, Kobe University, 1-1 Rokkodai, Nada-ku, Kobe, 657-8501 Japan; Department of Chemical Science and Engineering, Graduate School of Engineering, Kobe University, 1-1 Rokkodai, Nada-ku, Kobe, 657-8501 Japan; Department of Chemical Engineering, National Cheng Kung University, Tainan, 701 Taiwan; Research Center for Energy Technology and Strategy, National Cheng Kung University, Tainan, 701 Taiwan; Center for Bioscience and Biotechnology, National Cheng Kung University, Tainan, 701 Taiwan; Biomass Engineering Program, RIKEN, 1-7-22 Suehiro-cho, Tsurumi-ku, Yokohama, Kanagawa 230-0045 Japan

**Keywords:** Microalgae, *Chlamydomonas* sp, Lipids, Biodiesel, Metabolite profiling, Carbon flux, Light intensity, Nitrogen depletion

## Abstract

**Background:**

Marine microalgae are among the most promising lipid sources for biodiesel production because they can be grown on nonarable land without the use of potable water. Marine microalgae also harvest solar energy efficiently with a high growth rate, converting CO_2_ into lipids stored in the cells. Both light intensity and nitrogen availability strongly affect the growth, lipid accumulation, and fatty acid composition of oleaginous microalgae. However, very few studies have systematically examined how to optimize lipid productivity by adjusting irradiance intensity, and the metabolic dynamics that may lead to improved lipid accumulation in microalgae have not been elucidated. Little is known about the mechanism of lipid synthesis regulation in microalgae. Moreover, few studies have assessed the potential of using marine microalgae as oil producers.

**Results:**

In this work, a newly isolated marine microalga, *Chlamydomonas* sp. JSC4, was selected as a potential lipid producer, and the effect of photobioreactor operations on cell growth and lipid production was investigated. The combined effects of light intensity and nitrogen depletion stresses on growth and lipid accumulation were further explored in an effort to markedly improve lipid production and quality. The optimal lipid productivity and content attained were 312 mg L^−1^ d^−1^ and 43.1% per unit dry cell weight, respectively. This lipid productivity is the highest ever reported for marine microalgae. Metabolic intermediates were profiled over time to observe transient changes during lipid accumulation triggered by combined stresses. Finally, metabolite turnover was also assessed using an *in vivo*^13^C-labeling technique to directly measure the flow of carbon during lipid biosynthesis under stress associated with light intensity and nitrogen deficiency.

**Conclusions:**

This work demonstrates the synergistic integration of cultivation and dynamic metabolic profiling technologies to develop a simple and effective strategy for enhancing oil production in a marine microalga. The knowledge obtained from this study could be useful in assessing the feasibility of marine microalgae biodiesel production and for understanding the links between dynamic metabolic profiles and lipid biosynthesis during the course of microalgal cultivation.

**Electronic supplementary material:**

The online version of this article (doi:10.1186/s13068-015-0226-y) contains supplementary material, which is available to authorized users.

## Background

Due to the impact of the fast-growing global demand for fossil fuels on global climate change, considerable attention has been paid to reducing CO_2_ emissions by developing new sustainable energy sources as alternatives to fossil fuels [1]. Among the various potential renewable energy sources, biofuels produced from biomass are of the most interest and are expected to play an important role in the near future [2].

Biodiesel is an ideal renewable energy source because it is non-toxic and biodegradable and leads to lower CO_2_ emissions [3]. Microalgae-produced biodiesel is one of the most promising candidates, as some oleaginous microalgae can harvest solar energy efficiently due to their high growth rate and convert CO_2_ into lipids stored in the cells [4]. Compared with terrestrial plants, microalgae can grow 10 to 50 times faster, resulting in an extremely high CO_2_ fixation rate [5,6]. In addition, some oleaginous microalgae, such as *Chlorella*, *Chlamydomonas*, *Neochloris*, and *Dunaliella* species, can accumulate lipid up to 50% per unit dry weight of biomass, and the lipid composition is suitable for making biodiesel [7-10]. Furthermore, microalgal growth is not seasonally limited and can take place on nonarable land using municipal wastewater, brackish water, or seawater as a nutrient source [11,12]. The abovementioned characteristics make microalgal lipids promising raw materials for biodiesel synthesis.

Although microalgae seem to have a high potential for biodiesel production, obstacles that may hinder their industrial application remain. For instance, lipid accumulation in microalgae often occurs under environmental stress (for example, nitrogen depletion), which often leads to slower growth that results in lower lipid productivity [7,13]. Moreover, for large-scale microalgal cultivation, a tremendous amount of fresh water is required if the microalgae cannot grow well in brackish water, recycled water, or seawater. This may cause a shortage of available water resources, which are already very limited worldwide [7,14]. Therefore, cultivation strategies that result in optimal lipid content/productivity should be applied, and marine strains should be selected that are capable of rapid growth in saline environments and production of a high content of appropriate lipids.

To enhance the commercial feasibility of microalgae-based biodiesel production, the cultivation of lipid-producing microalgae must be made more efficient, with particular emphasis on optimization of several key factors that significantly affect the yield and properties of biodiesel, such as lipid productivity and composition. Recent studies have noted that suitable light intensity is very effective in promoting growth and lipid accumulation and for improving lipid quality in oleaginous microalgae [15,16]. However, no reports of systematic studies of how to optimize lipid productivity by adjusting irradiance intensity or studies profiling metabolite dynamics associated with improved lipid accumulation in microalgae have been published. In addition, relatively few studies examining the potential use of marine microalgae as oil producers have been published.

In oleaginous microalgae, lipids are synthesized from acetyl-CoA precursors during periods of light exposure through the actions of several enzymes composing the fatty acid synthesis pathway [17]. Although light stress and nitrogen depletion are known to strongly influence growth and lipid accumulation in microalgae [16,18,19], little is known regarding the mechanism of lipid synthesis regulation in microalgae. In addition, a thorough understanding of metabolite flux during lipid synthesis is also lacking.

Metabolic profiling is now a proven and powerful tool for gaining insights into functional biology [20,21]. The comprehensive analysis of a wide range of metabolites in microorganisms using mass spectrometry makes it possible to identify and quantify target metabolites that play key roles in specific biological pathways, thereby revealing the dynamic behavior of biological systems [20,22]. However, this metabolomic technology has yet to be widely applied to microalgae, particularly oleaginous microalgae [7]. To date, microalgal metabolomic studies have primarily focused on the metabolic responses of well-known species (for example, *Chlamydomonas reinhardtii*) to nitrogen/nutrient depletion or variations in CO_2_ concentration [23-25]. However, there have been no reports of the metabolic profiling of microalgae cultivated under the combined stress of deficiencies in irradiance and nitrogen availability.

Targeting metabolites using ^13^C-labeling technologies is another useful strategy for studying biological systems *in vivo* because it provides an overview of metabolite flux through different pathways [26]. However, most studies reported to date have merely obtained ‘snapshot’ metabolic profiles in microalgae; consequently, little is known about metabolic turnover or pathways in these organisms [22].

**Figure 1:**
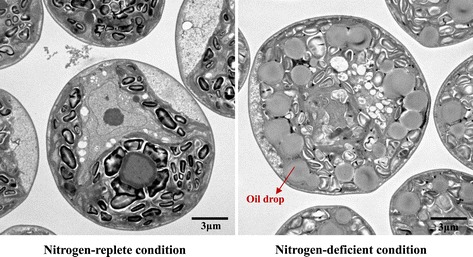
**Morphology of the oil body in** ***Chlamydomonas*** **sp. JSC4 cultivated under nitrogen-replete or nitrogen-deficient conditions (light intensity = 250 μmol m** ^**−2**^ **s** ^**−1**^ **; CO**_**2**_ **aeration = 2.0%; CO**_**2**_ **flow rate = 0.1 vvm).**

Salinity-tolerant microalgae capable of high growth rates and accumulation of high lipid content are rare in algae collection centers around the world. In this study, a newly isolated marine microalga, *Chlamydomonas* sp. JSC4, was used as a candidate lipid producer because the lipid content of this strain can reach over 45% to 50% of the dry cell weight (DCW) under environmental stress, as illustrated in Figure 1. Light supply is one of the most important factors affecting growth rate and lipid accumulation in phototrophic growth of microalgae [16,27]. Therefore, we initially explored the optimal cultivation conditions for microalgae-based biodiesel production by investigating the combined effects of photobioreactor (PBR) type, light intensity, and nitrogen depletion. The metabolic profile of *Chlamydomonas* sp. JSC4 under light effect and nitrogen depletion was also monitored to identify the intercorrelations between light intensity, nitrogen depletion, and lipid accumulation. Finally, metabolic turnover in *Chlamydomonas* sp. JSC4 was assessed using an *in vivo*^13^C-labeling technique to directly measure the flow of carbon during lipid biosynthesis under conditions of different light intensity and nitrogen deficiency. The aim of this work was to determine the optimal light intensity necessary to maximize the production of lipids of suitable composition for biodiesel production by analyzing the flux of metabolites in *Chlamydomonas* sp. JSC4. The knowledge obtained from this study could be useful in assessing the applicability of this strain as biodiesel feedstock and for enhancing understanding of metabolite dynamics during lipid biosynthesis under stress.

## Results and discussion

### Effect of PBR type on growth rate, CO_2_ fixation, and lipid accumulation in *Chlamydomonas* sp. JSC4

The growth rate and cellular composition of microalgae vary significantly with the illumination conditions during phototrophic growth [16]. In the PBRs used in our previous study [7], light intensity tended to decrease when the biomass concentration increased due to light-shielding effects, causing a significant decrease in growth rate and changes in cellular composition. Therefore, in this study, a new type of PBR was designed (designated a slim-type tubular PBR) to improve the light-receiving area of the PBR from 104.4 to 200.2 cm^2^. The target strain, *Chlamydomonas* sp. JSC4, was grown in both conventional and the newly designed PBRs illuminated with a one-side light intensity of 150 μmol m^−2^ s^−1^, with 2% CO_2_ serving as the carbon source.

**Figure 2:**
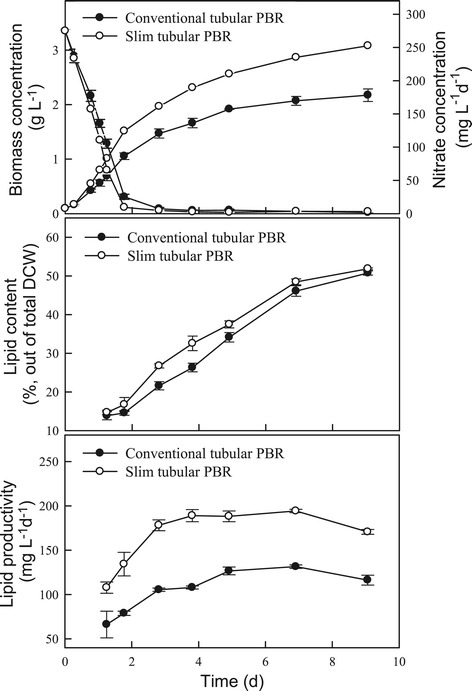
**Performance (growth, lipid content, and lipid productivity) of** ***Chlamydomonas*** **sp. JSC4 cultivated using different PBR types.** Error bars indicate standard deviation of three replicated experiments (light intensity = 150 μmol m^−2^ s^−1^; CO_2_ aeration = 2.0%; CO_2_ flow rate = 0.1 vvm).

As shown in Figure 2 and Table 1, the growth rate increased when using the slim-type tubular PBR, resulting in a higher CO_2_ fixation rate of 754 mg L^−1^ d^−1^, which was up to 40% higher than that achieved with a conventional-type tubular PBR. This phenomenon might have been due to the higher surface area to volume (S:V) ratio and shorter light path of the slim-type tubular PBR. The slim-type tubular PBR appears to have a greater light-receiving area and greater light penetration efficiency. Similar results were also observed in other research, demonstrating that a PBR with a high S:V ratio (80 to 100 m^2^ m^−3^) has a shorter light path and eliminates dark areas in the cell broth, thus leading to increased biomass concentration [28]. With respect to lipid production, Figure 2 shows that the type of PBR had no significant effect on the lipid content of *Chlamydomonas* sp. JSC4. Lipid productivity was thus positively proportional to biomass productivity. The maximum lipid productivity achieved with the slim-type tubular PBR was 194 mg L^−1^ d^−1^ (Table 1), which was significantly higher than that obtained with a conventional PBR. The above results indicate that the slim-type tubular PBR is more effective for growing strain JSC4, and thus this type or reactor was used in subsequent experiments.

**Table 1:** **Growth, lipid accumulation, and CO**_**2**_ **-fixation performance of marine** ***Chlamydomonas*** **sp. JSC4**

**PBR type**	**PBR diameter (cm)**	**S:V** ^**a**^ **ratio (m** ^**2**^ **m** ^**−3**^ **)**	**Light-receiving area (cm** ^**2**^ **)**	**Biomass productivity (mg L** ^**−1**^ **d** ^**−1**^ **)**	**Lipid content (%)**	**Lipid productivity (mg L** ^**−1**^ **d** ^**−1**^ **)**	**CO**_**2**_ **fixation rate (mg L** ^**−1**^ **d** ^**−1**^ **)**
Conventional	9.5	41.8	104.4	285 ± 13	46.1 ± 1.4	132 ± 2	537 ± 24
Slim	5.0	80.1	200.2	401 ± 10	48.5 ± 0.8	194 ± 2	754 ± 19

*Chlamydomonas* sp. JSC4 cultivated for 7 days (5 days of nitrogen depletion) in different photobioreactors (PBRs). Values are the mean ± standard deviation of three replicated experiments (light intensity = 200 μmol m^−2^ s^−1^; CO_2_ aeration = 2%; CO_2_ flow rate = 0.1 vvm).

^a^Surface area to volume ratio.

### Light intensity-dependent growth kinetics of *Chlamydomonas* sp. JSC4

**Figure 3:**
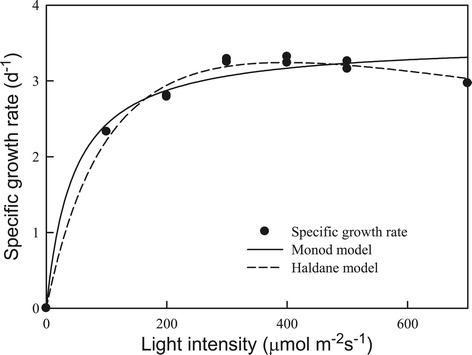
**Light intensity**-**dependent growth kinetics and model simulations of** ***Chlamydomonas*** **sp. JSC4 cultivated in a slim-type tubular PBR (CO**_**2**_ **aeration = 2.0%; CO**_**2**_ **flow rate = 0.1 vvm).**

For most photoautotrophic microalgae, the growth rate depends heavily on irradiation intensity [15,16]. Considering that *Chlamydomonas* sp. JSC4 was isolated from a coastal area in tropical Taiwan, it was thought that the light intensity of 150 μmol m^−2^ s^−1^ used in the experiments described above (Figure 2) would not be optimal for growth. Therefore, strain JSC4 was cultivated in the slim-type tubular PBR illuminated at different light intensities to determine the optimal light intensity. As shown in Figure 3 and Table 2, the specific growth rate of strain JSC4 increased rapidly as the light intensity increased from 100 to 300 μmol m^−2^ s^−1^, reaching a *plateau* at light intensities greater than 300 μmol m^−2^ s^−1^. The highest specific growth rate was around 3.2 d^−1^ in the light-saturation region (300 to 500 μmol m^−2^ s^−1^), which was significantly higher than that found in our previous work [7] and other related studies [15,16]. In addition, Table 2 shows that the specific growth rate of strain JSC4 at different light intensities could be fitted via Monod and Haldane models. The results fit the Haldane model with better agreement (*R*^*2*^ = 0.991), indicating that slight light inhibition may have occurred. However, due to the high K_I_ value of 1,097 μmol m^−2^ s^−1^, strain JSC4 appears to have a high tolerance to the inhibitory effects of high light intensity. These data indicate that strain JSC4 has good potential for outdoor culturing throughout the year in subtropical and tropical areas.

**Table 2:** **Estimated kinetic parameters of Monod- and Haldane-type models**

**Model**	**Estimated parameters**			
	**μ**_**max**_	**K**_**s**_	**K**_**I**_	***R*** ^***2***^
Monod	3.52	45.2	N.A.	0.972
Haldane	5.58	143.2	1,097	0.991

Based on the effect of light intensity on the growth of *Chlamydomonas* sp. JSC4 (CO_2_ aeration = 2%; CO_2_ flow rate = 0.1 vvm).

### Combined effects of light intensity and nitrogen depletion on growth, CO_2_ fixation, and lipid accumulation in *Chlamydomonas* sp. JSC4

To enhance the economic feasibility of microalgae-based biodiesel production, lipid synthesis should be maximized to reach a satisfactory lipid content. In addition, biomass productivity should be enhanced to ensure high lipid productivity. It is known that many microalgae can rapidly accumulate and store large amounts of lipids as an energy source in response to environmental stresses that are unfavorable to growth, such as high light intensity [19], nitrogen depletion [4,18], and high salinity [7]. Both salinity and nitrogen depletion have been shown to be critical factors that affect growth and lipid accumulation in microalgae. Our previous study indicated that cultivation of *Chlamydomonas* sp. JSC4 in 2% sea salt for 3 days under nitrogen-depletion conditions can enhance lipid productivity [7]. Furthermore, irradiance also strongly influences growth and lipid accumulation in some microalgae, including *Scenedesmus* [16], *Nannochloropsis* [19], *Pavlova* [29], and *Desmodesmus* [4]. As our previous study showed that strain JSC4 has a high potential to serve as an oil feedstock for biodiesel production [7], optimization of the irradiance conditions for this strain to achieve higher lipid productivity is of great importance. Obtaining a thorough understanding of the synergistic effects of light intensity control and nitrogen depletion is also very important. Another variable that must be evaluated is the cultivation time required to achieve the highest lipid production efficiency.

**Figure 4:**
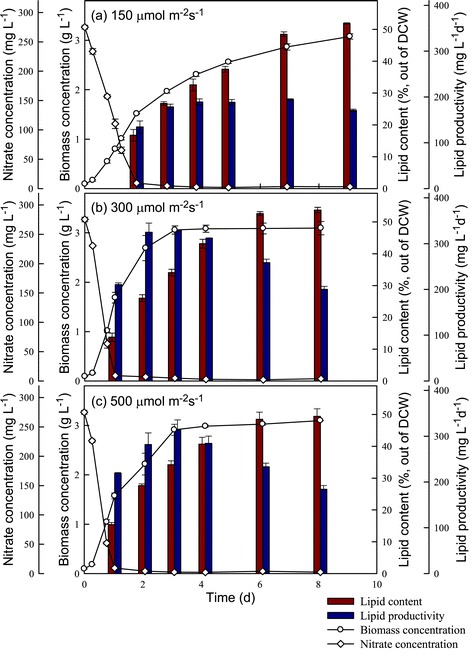
**Time-course profiles (a, b, c) of biomass concentration, nitrate concentration, lipid content, and lipid productivity during growth of** ***Chlamydomonas*** **sp. JSC4 illuminated with various light intensities.** Error bars indicate standard deviation of three replicated experiments (CO_2_ aeration = 2.0%; CO_2_ flow rate = 0.1 vvm).

**Figure 5:**
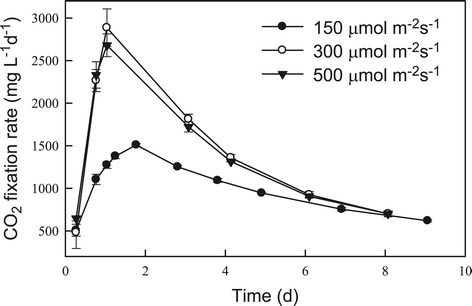
**Time-course profiles of CO**_**2**_ **fixation rate during the growth of** ***Chlamydomonas*** **sp. JSC4 illuminated with various light intensities.** Error bars indicate standard deviation of three replicated experiments (CO_2_ aeration = 2.0%; CO_2_ flow rate = 0.1 vvm).

In this study, strain JSC4 was cultivated under different light intensities for 8 to 9 days, with regular monitoring of biomass and nitrate concentrations, lipid content, lipid productivity, and CO_2_ fixation rate. As shown in Figures 4 and 5, the rates of growth and CO_2_ fixation increased significantly as the light intensity increased from 150 to 300 μmol m^−2^ s^−1^. However, a further increase in light intensity to 500 μmol m^−2^ s^−1^ resulted in a slight decrease in both the growth and CO_2_ fixation rates, suggesting that excessive illumination inhibits growth and CO_2_ fixation in this species, a phenomenon commonly known as the photo-inhibition effect. For instance, Ho *et al*. reported that under high light intensity (540 μmol m^−2^ s^−1^), the growth and CO_2_ fixation rates in *Scenedesmus obliquus* decline markedly [16]. In our experiments, a maximum CO_2_ fixation rate of 2,886 mg L^−1^ d^−1^ was obtained at a light intensity of 300 μmol m^−2^ s^−1^ during the optimal time period indicated in Figure 5. This performance was superior to that obtained both in our previous study [7] and that of most related studies [6]. This excellent CO_2_ fixation performance makes strain JSC4 a potential candidate for use in microalgae-based CO_2_ mitigation processes for real industrial fuel gases.

With respect to lipid production in strain JSC4, Figure 4 shows that lipid productivity increased slightly as the light intensity increased from 150 to 300 μmol m^−2^ s^−1^, due primarily to enhanced growth associated with the increase in light intensity. In addition, previous reports showed that high light intensity may lead to an increase in the amount of neutral storage lipids, primarily triacylglycerol (TAG) derived from fatty acids (referred to as lipids in this study) [17,29]. However, in our study, a further increase in light intensity to 500 μmol m^−2^ s^−1^ resulted in a slight drop in lipid content (Figure 4), indicating that excess light intensity inhibits lipid biosynthesis. Moreover, at all light intensities we examined, lipid content increased significantly during stationary phase (during which nitrogen deficiency occurred), suggesting that lipid accumulation in strain JSC4 is not only triggered by the supply of irradiance but also by the stress of nitrogen depletion. Pal *et al*. [19] also demonstrated that exposing microalgae to more than one stress simultaneously (for example, high salinity and nitrogen depletion) may be necessary for further enhancement of lipid accumulation. Therefore, as shown in Figures 4 and 5, appropriately increasing the light intensity to promote growth in combination with limiting the nitrogen supply to trigger lipid accumulation can lead to a simultaneous increase in both the lipid content and productivity in strain JSC4. At a light intensity of 300 μmol m^−2^ s^−1^, strain JSC4 displayed a maximum lipid productivity of 328 mg L^−1^ d^−1^ and lipid content of 34.1%.

From an engineering perspective, lipid productivity is absolutely one of the most important performance indicators for evaluating the commercialization potential of lipid-producing microalgae. Moreover, the lipid content is also important when considering the operation costs of downstream lipid extraction and purification processes [16]. Normally, the lipid content of microalgae tends to increase when the nitrogen starvation period is extended, until the maximum lipid content is reached. However, such a prolonged cultivation time leads to a decrease in lipid productivity. Therefore, it was vital to determine the optimal incubation time for strain JSC4 under nitrogen depletion as well as the timing of cell harvesting to ensure the best lipid production performance. At the optimal light intensity determined in this study (300 μmol m^−2^ s^−1^), the lipid content increased sharply as the nitrogen depletion time increased, until reaching a maximum value of 53.7% after 7 days (Table 3). However, it is difficult to optimize both lipid content and lipid productivity simultaneously, because during the nitrogen depletion period required for triggering lipid accumulation, growth appeared to slow, leading to lower lipid productivity (Table 3). Therefore, based on these results, optimal lipid production (lipid content = 43.1%; lipid productivity = 312 mg L^−1^ d^−1^) was obtained at a light intensity of 300 μmol m^−2^ s^−1^ and a 3-day nitrogen depletion period. The resulting lipid productivity of 312 mg L^−1^ d^−1^ was significantly higher than most of the values reported in the literature, as indicated in Table 4.

**Table 3:** **The effects of duration of nitrogen depletion on lipid content and lipid productivity**

**Duration of N depletion (d)**	**Lipid content (% of DCW)**	**Lipid productivity (mg L** ^**−1**^ **d** ^**−1**^ **)**
0	13.8 ± 1.3	210 ± 4
1	26.0 ± 1.1	325 ± 20
2	34.1 ± 1.1	328 ± 1
3	43.1 ± 1.4	312 ± 2
5	52.6 ± 0.6	259 ± 8
7	53.7 ± 0.8	200 ± 7

*Chlamydomonas* sp. JSC4 cultured at a light intensity of 300 μmol m^−2^ s^−1^ (CO_2_ aeration = 2.0%; CO_2_ flow rate = 0.1 vvm.).

**Table 4:** **Comparison of lipid content and lipid productivity**

**Species**	**Lipid content (%)**	**Lipid productivity (mg L** ^**−1**^ **d** ^**−1**^ **)**	**Reference**
*Chlorella sorokiniana* CY1	57.7	140.8	[2]
*Chlorella vulgaris*	17.3	79.1	[31]
*Chlorella emersonii*	18.6	54.4	[31]
*Chlorella protothecoides*	43.4	N.A.	[46]
*Chlamydomonas* sp. JSC4	33.1	169.1	[47]
*Chlamydomonas* sp. JSC4	59.4	223	[7]
*D. tertiolecta* UTEX LB999	25.9	N.A.	[48]
*D. tertiolecta* ATCC30929	67.0	N.A.	[49]
*Isochrysis zhangjiangensis*	46.0	136.2	[50]
*Nannochloropsis* sp.	31.3	N.A.	[27]
*Nannochloropsis* sp.	59.9	74.2	[14]
*Nannochloropsis* sp. F&M-M24	68.5	110.1	[51]
*Nannochloropsis gaditana*	73.1	51	[52]
*Nannochloropsis* sp. F&M-M28	35.7	60.9	[53]
*Nannochloropsis* sp. F&M-M26	29.6	61.0	[53]
*T. pseudonana* CCMP1335	20.3	N.A.	[48]
*Tetraselmis suecica* F&M-M33	39.1	53.1	[51]
*Chlamydomonas* sp. JSC4	43.1	312	This study

Compared *Chlamydomonas* sp. JSC4 with that of other marine microalgae as reported in the literature.

N.A., not available.

### The effect of light intensity on lipid quality in *Chlamydomonas* sp.

In addition to lipid content and productivity, the fatty acid composition is also important because it provides an indication of a strain’s suitability for synthesizing biodiesel [16]. The fatty acid composition of phototrophic microalgae often changes under different irradiance conditions [17]. In this study, the lipid composition of *Chlamydomonas* sp. JSC4 was monitored with respect to light intensity. As shown in Table 5, the major fatty acids present in the lipids produced by strain JSC4 were similar when the cells were cultivated under different light intensities. The predominant fatty acids were palmitic acid (27% to 30%), stearic acid (14% to 15%), oleic acid (22% to 25%), and linoleic acid (16% to 19%). However, the total amount of C16/C18 fatty acids increased slightly, from 90.0% to 92.5%, when the light intensity was increased. Similar results were also reported by Sun *et al*. [18], who noted that the fatty acids with 16- and 18-carbon atoms in *Neochloris oleoabundans* HK-129 increased as the light intensity increased from 50 to 200 μmol m^−2^ s^−1^. In addition, Table 5 shows that the proportions of the main saturated (for example, palmitic and stearic) and monounsaturated (for example, palmitoleic and oleic) fatty acids increased slightly as light intensity increased, indicating that high light intensity alters fatty acid biosynthesis in microalgae, resulting in the production of more of the saturated or monounsaturated fatty acids that primarily make up neutral lipids [17]. These phenomena were also observed in other studies demonstrating that high light intensity triggers the biosynthesis of palmitic, setaric, or oleic acids in microalgae. This lipid composition appears to be adequate for producing biodiesel [18,30].

**Table 5:** **Fatty acid composition of** ***Chlamydomonas*** **sp. JSC4**

	***Chlamydomonas*** **sp. JSC4 FA composition**		
	**150 μmol m** ^**−2**^ **s** ^**−1**^	**300 μmol m** ^**−2**^ **s** ^**−1**^	**500 μmol m** ^**−2**^ **s** ^**−1**^
Palmitic acid (C16:0)	27.3 ± 0.4	28.5 ± 0.6	29.5 ± 0.5
Palmitoleic acid (C16:1)	2.0 ± 0.2	2.5 ± 0.1	3.1 ± 0.2
Stearic acid (C18:0)	14.0 ± 0.3	14.9 ± 0.5	15.4 ± 0.3
Oleic acid (C18:1)	22.0 ± 0.1	22.9 ± 0.4	24.5 ± 0.9
Linoleic acid (C18:2)	18.7 ± 0.2	18.5 ± 0.3	15.5 ± 0.3
Linolenic acid (C18:3)	5.9 ± 0.1	5.1 ± 0.1	4.9 ± 0.2
Saturated fatty acid	41.9 ± 0.5	43.4 ± 0.9	44.9 ± 0.7
Monounsaturated fatty acid	24.0 ± 0.2	25.4 ± 0.4	27.6 ± 0.9
Polyunsaturated fatty acid	24.6 ± 0.2	23.6 ± 0.4	20.4 ± 0.4
C16 and C18 groups	90.0 ± 1.4	92.3 ± 1.8	92.5 ± 2.1
Lipid content (%)	48.5 ± 0.8	53.1 ± 1.3	48.9 ± 2.8

*Chlamydomonas* sp. JSC4 cultured for 5 days under nitrogen depleted conditions and different light intensities. Values are the mean ± standard deviation of three replicated experiments (CO_2_ aeration = 2.0%; CO_2_ flow rate = 0.1 vvm.).

The effect of light intensity on five important properties (namely, kinematic viscosity, specific gravity, cetane number, iodine number, and average unsaturation) of biodiesel produced from the oil from strain JSC4 is summarized in Table 6. Because an increase in the degree of saturation of the oil used in biodiesel production can increase the cetane number and oxidative ability, whereas a higher degree of unsaturation can improve the fluidity of biodiesel at low temperatures [31,32], the average degree of unsaturation appears to be an essential indicator of biodiesel quality [32]. As shown in Table 6, although the biodiesel obtained from strain JSC4 grown under high light intensity had a slightly lower average degree of unsaturation, the lipid characteristics (that is, kinematic viscosity, specific gravity, cetane number, and iodine number) were not significantly affected by the illumination conditions, as all of these properties showed satisfactory values based on the United States biodiesel standard ASTM D6751 and the European EN 14214 standard. Thus, regardless of the light intensity, the lipids produced by *Chlamydomonas* sp. JSC4 appeared to be of adequate quality for biodiesel synthesis.

**Table 6:** **Biodiesel properties of** ***Chlamydomonas*** **sp. JSC4**

**Biodiesel property**	**150 μmol m** ^**−2**^ **s** ^**−1**^	**300 μmol m** ^**−2**^ **s** ^**−1**^	**500 μmol m** ^**−2**^ **s** ^**−1**^	**US standard (ASTM D6751-08)**	**European standard (EN 14214)**
Kinematic viscosity^a^	4.71	4.72	4.74	1.9 to 6.0	3.5 to 5.0
Specific gravity^b^	0.88	0.88	0.88	0.85 to 0.90	0.86 to 0.90
Cetane number^c^	57.60	57.69	57.99	min47	min51
Iodine number^d^	71.54	70.53	67.24	-	max120
Average unsaturation	0.79	0.78	0.73	-	-

The fatty acids produced from dried biomass of *Chlamydomonas* sp. JSC4 cultured for 5 days under conditions of nitrogen depletion and different light intensities. The relationship between average degree of unsaturation of fatty acids and biodiesel properties as reported by Song *et al*. [32] are shown. Values are the mean of three replicated experiments (CO_2_ aeration = 2.0%; CO_2_ flow rate = 0.1 vvm.).

^a^Calculated from the following equation: Kinematic viscosity (mm^2^ s^−1^, at 40°C) = ^−^0.6316 × average unsaturation + 5.2065.

^b^Calculated from the following equation: Specific gravity (kg L^−1^) = 0.0055 × average unsaturation + 0.8726.

^c^Calculated from the following equation: Cetane number = ^−^6.6684 × average unsaturation + 62.876.

^d^Calculated from the following equation: Iodine number (gl_2_ g^−1^) = 74.373 × average unsaturation + 12.71.

### Effect of specific light availability on lipid content and lipid production in *Chlamydomonas* sp. JSC4

**Figure 6:**
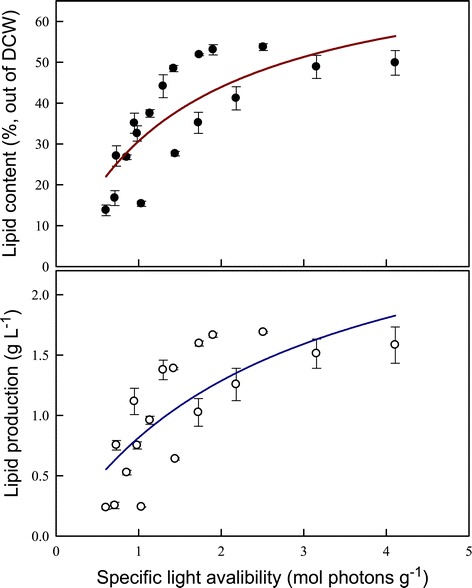
**The effect of specific light availability on lipid accumulation over time in** ***Chlamydomonas*** **sp. JSC4.** Error bars indicate standard deviation of three replicated experiments (CO_2_ aeration = 2.0%; CO_2_ flow rate = 0.1 vvm).

To further evaluate the feasibility of biodiesel production using *Chlamydomonas* sp. JSC4, the influence of differences in specific light availability on lipid production was investigated. Specific light availability was defined as moles of photons per gram DCW in the PBR over a designated time interval [33]. According to Figure 6, both lipid content and lipid production were positively correlated with the specific light availability. The data clearly show that both lipid content and lipid production increase dramatically initially when the specific light availability is increased until reaching a maximal value. It has been reported that the total amount of energy bound per gram of biomass increases with increasing lipid content because the heating value of the microalgal biomass increases from 18.8 to 27.1 MJ kg^−1^ as the lipid content increases from 10% to 50% [33]. Wilhelm *et al*. [34] also reported that the photon demand increases during lipid biosynthesis in microalgae relative to the demand for primary sugar biosynthesis because of the additional reduction steps. Therefore, an increase in light energy provides more energy for both growth and lipid biosynthesis. However, as shown in Figure 6, increasing the specific light availability to 3 to 4 mol photons g^−1^ results in a slight drop in both lipid content and lipid production, suggesting that the excess illumination inhibits photosynthetic efficiency and lipid synthesis in microalgae [35]. Thus, specific light availability is a simple but important indicator of the relationship between light supply and lipid production in microalgae that have the potential for large-scale outdoor culture.

### Metabolic profiling of *Chlamydomonas* sp. JSC4 under light stress and nitrogen depletion

Lipid accumulation in microalgae is often affected by environmental stimuli, acting either individually or in combination [17]. Some researchers have postulated that exposing microalgae to combined stresses (such as simultaneous high salinity and nitrogen depletion) may further enhance lipid production efficiency [7,19]. However, little is known about the metabolic details of lipid biosynthesis in response to different light intensities used for cell growth. Metabolic profiling techniques appear to be well-suited for studies aimed at elucidating the mechanisms underlying metabolic changes in lipid biosynthesis in microalgae exposed to various environmental stimuli (such as irradiance and nitrogen deficiency) [23]. To the best of our knowledge, this is the first report demonstrating the metabolic profile of microalgae cultivated under combined light irradiation and nitrogen depletion.

**Figure 7:**
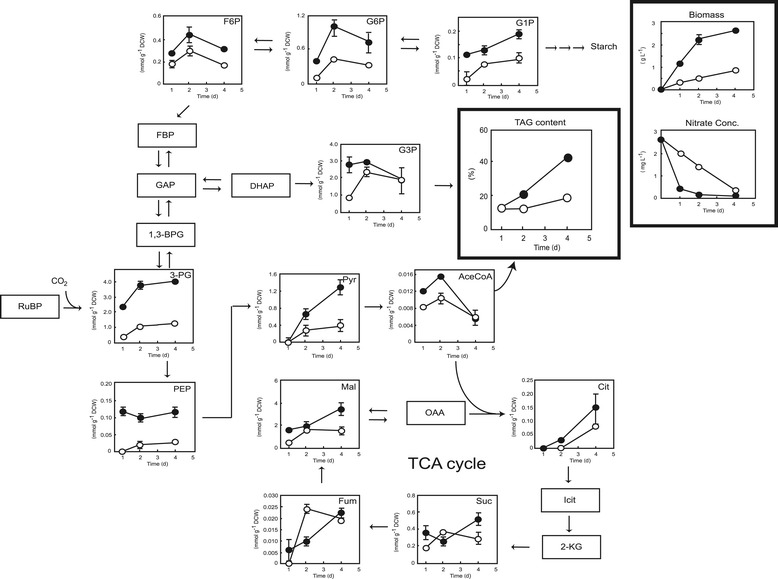
**Time-course analysis of metabolite content of** ***Chlamydomonas*** **sp. JSC4 cells illuminated with high light intensity (300 μmol m** ^**−2**^ **s** ^**−1**^ **; closed circles) or low light intensity (30 μmol m** ^**−2**^ **s** ^**−1**^ **; open circles).** Error bars indicate standard deviation of three replicated experiments (CO_2_ aeration = 2.0%; CO_2_ flow rate = 0.1 vvm). Abbreviations: AceCoA, acetyl-CoA; 1,3-BPG, 1,3-bisphosphoglycerate; Cit, citrate; DHAP, dihydroxyacetone phosphate; FBP, fructose-1,6-bisphosphate; F6P, fructose-6-phosphate; Fum, fumarate; GAP, glyceraldehyde-3-phosphate; G1P, glucose-1-phosphate; G3P: glycerol-3-phosphate; G6P, glucose-6-phosphate; 2-KG, 2-ketoglutarate; Mal, malate; OAA, oxaloacetate; PEP, phosphoenolpyruvate; 3-PG, 3-phosphoglycerate; Pyr, pyruvate; RuBP, ribulose; Suc, succinate; TAG, triacylglycerol.

In this study, *Chlamydomonas* sp. JSC4 was cultivated under low and high light intensities (30 and 300 μmol m^−2^ s^−1^), respectively. Intracellular metabolites were extracted and analyzed using capillary electrophoresis coupled with mass spectrometry (CE/MS), and variations in metabolite amounts over time were monitored as described previously [7]. As shown in Figure 7, cultivation at high light intensity resulted in a higher nitrogen consumption rate and a higher biomass concentration compared with cells cultured under low light intensity, suggesting that 300 μmol m^−2^ s^−1^ is a more suitable light intensity for lipid accumulation in *Chlamydomonas* sp. JSC4. Thus, optimization of the light intensity provides a direct means of obtaining higher cell densities and enhanced lipid content. Notably, the levels of TAG-synthesis-related metabolites (for example, glycerol-3-phosphate [G3P] and acetyl-CoA (AceCoA)) in cells cultured under high light intensity were significantly higher than in cells cultured under low light intensity. This result is in good agreement with previous reports showing that irradiation with high-intensity light provides more energy during cultivation, which may induce microalgae to accumulate more TAG as the neutral storage lipid [17,36]. Moreover, it is known that the photon demand increases during lipid synthesis due to the additional reduction steps [34], and the metabolites of both G3P and AceCoA are extremely important precursors for triacylglycerol formation [17,37]. The positive relationship between G3P, AceCoA, and triacylglycerol synthesis observed in this study in strain JSC4 cultured under high light intensity was therefore expected. Sufficient light supply thus clearly triggers the formation of G3P and AceCoA and then further promotes the synthesis of TAG.

The levels of several key metabolites involved in the Calvin cycle (3-phosphoglyceric acid (3-PG), fructose-6-phosphate (F6P), and glucose-6-phosphate (G6P)) increased significantly along with cell growth, reaching maximal levels on day 2. This suggests that nutrients such as nitrogen either in the medium or inside the cells are sufficient for cell viability. However, after 2 days of nitrogen limitation (day 4), the levels of several metabolites involved in the Calvin cycle (F6P and G6P) decreased, primarily due to transformation of energy compounds into TAG (Figure 7). Moreover, regardless of nitrate status, an increase in the level of the starch precursor glucose-1-phosphate (G1P) was observed in cells irradiated with high-intensity light. This result indicates that strain JSC4 may also store considerable amounts of starch as an energy compound when exposed to certain environmental conditions. Previous reports showed that high light intensity may lead to an increase in the amount of total carbohydrate at microalga *S. obliquus*, which is in good agreement with our study. In addition, several studies have shown that competition between lipid and carbohydrate synthesis occurs in microalgae under stress conditions [38,39]. Therefore, blocking the carbohydrate synthesis pathway may provide an approach for boosting TAG production. Furthermore, in the present study, cultivation under high light intensity led to a dramatic accumulation of intermediates involved in glycolysis, such as 3-PG, phosphoenolpyruvate (PEP), and pyruvate (Pyr). This may have been caused by rate-limiting conversions (that is, conversion of Pyr to AceCoA) that restricted the accumulation of lipids.

CO_2_ is assimilated through the Calvin cycle in a reaction catalyzed by ribulose-1,5-biphosphate carboxylase/oxygenase (Rubisco) to form 3-PG. Under low light intensity, the pool size (intracellular concentration) of 3-PG remained low (Figure 7), perhaps because insufficient light energy was available to drive photosynthesis, thus further restricting the growth and ultimately the biosynthesis of lipids.

### Analysis of metabolic turnover in *Chlamydomonas* sp. JSC4 under light stress and nitrogen depletion

As shown in Figure 4, cultivation under the dual effects of light irradiation and nitrogen depletion led to a marked increase in lipid content in *Chlamydomonas* sp. JSC4. Metabolic profiling using CE/MS enables the identification of key metabolites of interest and provides a ‘snapshot’ of the metabolic status of cells at a given point in time. To further understand the dynamics of metabolic flux in JSC4 cells, CE/MS analysis combined with *in vivo* isotope labeling was employed [40]. Recent studies showed that the metabolic kinetics of microalgae can be estimated by measuring changes in the abundance of isotopomers over time [20,22,26].

In the present study, metabolic turnover, defined as the change in the ratio of carbon that is newly incorporated into metabolites to total metabolic carbon, was determined in the presence and absence of nitrate under different light intensities using an *in vivo*^13^C-labeling assay. The ratio of ^13^C to total carbon in each metabolite (that is, the ^13^C fraction (%)) was calculated from mass isotopomer distributions determined by MS. By determining the ratio of the intensity of the monoisotopic ion to that of its isotopic ions, the ratio of stable-isotope-labeled to unlabeled metabolites can be determined. In *in vivo*^13^C-labeling, ^13^C is assimilated through the Calvin cycle enzyme Rubisco in the form of ^13^CO_2_, which is then used in the production of 3-PG. In the presence of nitrate, the fraction of ^13^C associated with 3-PG reached a maximum of 80% after 30 min of labeling in cells cultured under high light intensity (300 μmol m^−2^ s^−1^), but it reached only 65% in cells cultured under low light intensity (30 μmol m^−2^ s^−1^). Irradiation with high light intensity also resulted in an increase in the ^13^C-labeling ratio for other sugar phosphates, such as F6P, G6P, G1P, G3P, PEP, and Pyr. These results indicate that sufficient irradiation leads to increases in both biomass concentration and lipid content, as shown in Figures 4 and 6. The ^13^C fraction did not reach 100% for any metabolites in this study. This result was in agreement with previous reports showing that the ^13^C fraction rarely reaches 100% for any metabolite in *Arhrospira platensis*, *Nicotiana tabacum*, and *Quercus rubra* [20,41]. This phenomenon may be the result of competition between isotope-labeled carbon and carbon derived from internal storage for assimilation into metabolites ultimately reaching an equilibrium phase.

**Figure 8:**
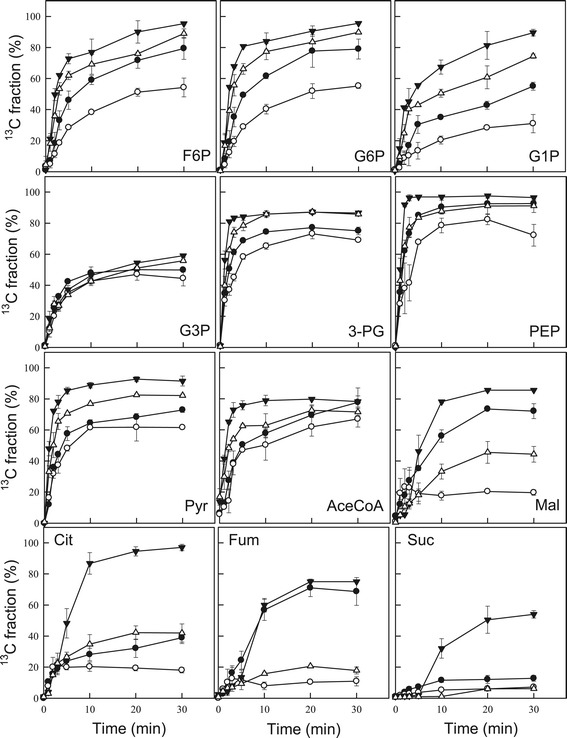
**Time-course analysis of the metabolite** ^**13**^ **C fraction of** ***Chlamydomonas*** **sp. JSC4 cells cultivated under high light intensity of 300 μmol m** ^**−2**^ **s** ^**−1**^ **, nitrogen-rich (closed triangles) conditions; high light intensity of 300 μmol m** ^**−2**^ **s** ^**−1**^ **, nitrogen-free (open triangles) conditions; low light intensity of 30 μmol m** ^**−2**^ **s** ^**−1**^ **, nitrogen-rich (closed circles) conditions; or low light intensity of 30 μmol m** ^**−2**^ **s** ^**−1**^ **, nitrogen-free (open circles) conditions.** Error bars indicate standard deviation of three replicated experiments. Abbreviations: AceCoA, acetyl-CoA; Cit, citrate; F6P, fructose-6-phosphate; Fum, fumarate; G1P, glucose-1-phosphate; G3P: glycerol-3-phosphate; G6P, glucose-6-phosphate; Mal, malate; PEP, phosphoenolpyruvate; 3-PG, 3-phosphoglycerate; Pyr, pyruvate; Suc, succinate.

Under conditions of nitrate depletion, the highest value of the ^13^C fraction decreased for most metabolites. Notably, by limiting the nitrate supply at a fixed light intensity of 300 μmol m^−2^ s^−1^, the ^13^C fractions of malate, citrate, fumarate, and succinate declined from 85.6%, 97.1%, 75.0%, and 54.0% to 45.6%, 42.2%, 20.6%, and 6.2%, respectively, suggesting that availability of ^13^C for use in the TCA cycle declines when cells are confronted with nitrogen starvation, despite an increase in lipid content (Figure 8). Similar phenomena were also observed in cells cultured under low light intensity (30 μmol m^−2^ s^−1^). It is not clear whether these responses are common for *Chlamydomonas* sp. and other photosynthetic species. However, several recent reports demonstrated that cultivation under nitrogen-deficient conditions leads to a decrease in turnover of TCA cycle intermediates [20], which is consistent with our findings.

**Figure 9:**
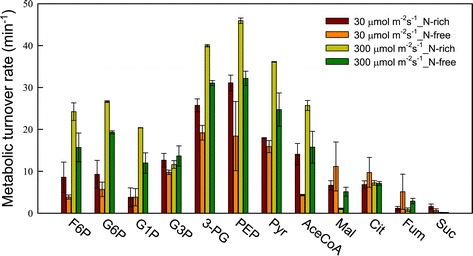
**Metabolic turnover rates of key metabolites in** ***Chlamydomonas*** **sp. JSC4 cells cultivated under high light intensity of 300 μmol m** ^**−2**^ **s** ^**−1**^ **, nitrogen-rich (yellow bars) conditions; high light intensity of 300 μmol m** ^**−2**^ **s** ^**−1**^ **, nitrogen-free (green bars) conditions; low light intensity of 30 μmol m** ^**−2**^ **s** ^**−1**^ **, nitrogen-rich (red bars) conditions; or low light intensity of 30 μmol m** ^**−2**^ **s** ^**−1**^ **, nitrogen-free (orange bars) conditions.** Error bars indicate standard deviation of three replicated experiments. Abbreviations: AceCoA, acetyl-CoA; Cit, citrate; F6P, fructose-6-phosphate; Fum, fumarate; G1P, glucose-1-phosphate; G3P: glycerol-3-phosphate; G6P, glucose-6-phosphate; Mal, malate; PEP, phosphoenolpyruvate; 3-PG, 3-phosphoglycerate; Pyr, pyruvate; Suc, succinate.

The initial slope of a plot of ^13^C fraction versus time provides an estimate of the turnover rate of each metabolite, as depicted in Figure 9. The turnover rates of sugar phosphates involved in the Calvin cycle (for example, PEP and 3-PG) were significantly higher than those of organic acids, confirming that in photosynthetic microorganisms, 3-PG is rapidly generated through the action of the Calvin cycle and then further converted to PEP and other intracellular metabolites (Figure 7). Similar trends were also reported by Hasunuma *et al*. [20], who found that the turnover rates of sugar phosphates are significantly higher than those of organic acids and amino acids in *A. platensis*. In addition, we observed that an increase in light intensity from 30 to 300 μmol m^−2^ s^−1^ led to a sharp rise in the turnover rate of most metabolites (for example, Pyr and AceCoA), which is consistent with the efficient growth of cells and accumulation of lipids demonstrated in Figures 4 and 6. Furthermore, under nitrogen limitation, the turnover rate of most metabolites in strain JSC4 decreased, suggesting that under nitrogen depletion, more energy is required for lipid synthesis, thus restricting the rate of carbon flow. Notably, the pool size of AceCoA (0.01 to 0.03 nmol mg^−1^), one of the most important TAG-synthesis-related metabolites, was approximately 20 to 50 times smaller than that of Pyr (0.32 to 0.78 nmol mg^−1^) (as shown in Additional file 1: Table S1), suggesting that lipid accumulation in strain JSC4 could be enhanced further through overexpression of related enzymes. Some efforts have been made to increase the expression of enzymes that are involved in fatty acid biosynthesis pathways, and the results are quite promising [42,43]. As a next step, knockout and overexpression of the genes involved in lipid synthesis could clarify which genes have a significant effect on lipid accumulation and lead to the development of strategies for increasing the lipid content in oleaginous microalgae.

Nitrogen limitation is frequently used to increase the lipid content in *Chlamydomonas* sp. [7]. However, little is known about the mechanism of lipid biosynthesis in these microorganisms. To the best of our knowledge, this is the first report of *in vivo* isotope labeling of *Chlamydomonas* sp. cultivated under different light-intensity conditions coupled with nitrogen limitation. In this study, the combination of metabolic profiling and turnover analysis (collectively referred to as dynamic metabolic profiling) was applied to explore the effects of stress associated with combined light intensity and nitrogen depletion on carbon metabolism in *Chlamydomonas* sp. JSC4. The outcome of this research enhanced current understanding of the relationship between carbon flow and lipid biosynthesis in microalgae cultured under combined light effect and nitrogen depletion. Using the *in vivo*^13^C-labeling assay, the dynamic metabolic profile of strain JSC4 was determined, generating a large amount of valuable information that can be applied to enhance the lipid production capacity of this organism through genetic engineering.

## Conclusions

This work demonstrated that lipid production in the marine microalga *Chlamydomonas* sp. JSC4 can be enhanced significantly by culturing the organism under conditions of appropriate light intensity (that is, 300 μmol m^−2^ s^−1^) coupled with nitrogen depletion. The highest lipid productivity achieved in this study was 328 mg L^−1^ d^−1^, which to the best of our knowledge is the highest value reported in the literature for marine microalgae. The lipids produced under the optimal conditions contained 43.4% saturated, 25.4% monounsaturated, and 23.6% polyunsaturated fatty acids, a composition very suitable for biodiesel production. The data obtained from dynamic metabolic profiling (that is, metabolic profiling and turnover analysis) provided valuable information that increases our understanding of metabolic flow in *Chlamydomonas* sp. JSC4 when cultured in the absence of nitrate and with light of suitable intensity. By integrating cultivation strategies and dynamic metabolic profiling techniques using strain JSC4, this study demonstrated an innovative technology for practically and markedly enhancing microalgal lipid productivity and elucidated the details of carbon metabolism related to lipid biosynthesis.

## Methods

### Microalgal strain and preculturing medium

The microalga used in this study was *Chlamydomonas* sp. JSC4, which was isolated from a coastal area of southern Taiwan. The 18S rDNA sequence of this strain was deposited in the National Center for Biotechnology Information GenBank database under accession number KF383270. The medium used for preculturing strain JSC4 was Modified Bold 3 N medium (4.4 mM NaNO_3_, 0.22 mM K_2_HPO_4_, 0.3 mM MgSO_4_ · 7H_2_O, 0.17 mM CaCl_2_ · 2H_2_O, 0.43 mM KH_2_PO_4_, and 0.43 mM NaCl; the levels of metals in the medium are described in Berges *et al*. [44]). Cells were routinely cultured at 30°C to 33°C for 3 to 4 days with a continuous supply of 2.0% CO_2_ at an aeration rate of 0.05 vvm. The culture was illuminated continuously at a light intensity of approximately 250 μmol m^−2^ s^−1^. The light intensity was measured using a Li-250 Light Meter with a Li-190SA pyranometer sensor (Li-COR Inc., Lincoln, NE, USA).

### PBR operation

Both conventional-type (9.5 cm in diameter; height to diameter (H:D) ratio of 0.8) and slim-type (5.0 cm in diameter; H:D ratio of 10) PBRs equipped with external light sources were used in this study. The S:V ratio of the conventional and slim-type PBRs was 41.8 and 80.1, respectively. *Chlamydomonas* sp. JSC4 was precultured in Modified Bold 3 N medium and inoculated into the PBRs at a concentration of 90 to 100 mg L^−1^. For batch cultures, microalgae were cultivated at 32°C to 35°C and pH 6.5 to 7.5 with an agitation rate of 400 rpm under various light intensities in the range of approximately 30 to 500 μmol m^−2^ s^−1^. As the sole carbon source, 2.0% CO_2_ was continuously fed into the culture at a rate of 0.05 vvm. Liquid samples were collected from the sealed glass vessel at designated time intervals to determine the culture density, pH, and residual nitrate concentration. The amount of CO_2_ reduced was determined by measuring the difference between the CO_2_ concentrations in the influent and effluent streams of the PBR using a GM70 CO_2_ detector (Vaisala, Tokyo, Japan).

### Determination of culture density

The density of microalgae in the PBR was determined regularly by measuring the optical density (OD) at a wavelength of 682 nm (denoted as OD_682_) using a UVmini-1240 UV/Vis spectrophotometer (Shimadzu, Kyoto, Japan) after proper dilution with deionized water to give a range of measurement between 0.1 and 0.9. The DCW was determined by filtering 50-mL aliquots of culture through a cellulose acetate membrane filter (0.45-μm pore size, 47 mm diameter). Each loaded filter was then freeze-dried until the weight was invariant. The dry weight of a blank filter was subtracted from that of the loaded filter to obtain the DCW of microalgae. The OD_682_ values were also converted to biomass concentration via appropriate calibration between OD_682_ and DCW, and the conversion factor was determined as 1.0 OD_682_ = 0.7 ~ 0.9 g DCW L^−1^.

### Measurement of residual nitrate content

Nitrate concentration was determined according to our previously reported method [45]. In general, each sample collected from the PBR was filtered with a 0.22-μm pore-size filter and then diluted 20-fold with deionized water. The residual nitrate content of the diluted samples was determined according to the OD_220_ with appropriate calibrations. The conversion factor was determined as 1.0 OD_220_ = ~389.3 mg nitrate L^−1^.

### Determination of growth parameters and CO_2_ fixation performance

Change in biomass concentration (mg L^−1^) over time was used to calculate the specific growth rate (d^−1^) based on a plot of DCW (on a logarithmic scale) versus time. Biomass productivity (mg L^−1^ d^−1^) was calculated according to the following equation:

$$ P=\frac{\Delta X}{\Delta t}, $$

where Δ*X* represents the variation in biomass concentration (mg L^−1^) over cultivation time (d).

The CO_2_ fixation rate (*P*_CO2_; mg L^−1^ d^−1^) was calculated according to the following equation:

$$ {P}_{{\mathrm{CO}}_2}\left(\mathrm{mg}/\mathrm{L}/\mathrm{d}\right)=1.88\times {P}_{\mathrm{biomass}}, $$

where *P*_biomass_ represents biomass productivity (mg L^−1^ d^−1^), as described above. The typical molecular formula for microalgal biomass (CO_0.48_H_1.83_N_0.11_P_0.01_) [10] was used in this study.

To evaluate the effect of irradiance along with biomass concentration on the lipid accumulation process, the specific light availability (*I*_PAR, spec_) as reported by Munkel *et al*. [33] was calculated according to the following equation:

$$ {I}_{\mathrm{PAR},\;\mathrm{spec}}=\frac{{\displaystyle \int }{I}_{\mathrm{PAR}\;}dt}{\varnothing \mathrm{D}\mathrm{W}\mathrm{V}}, $$

where *I*_PAR_ represents irradiance on the PBR surface integrated over a defined time interval. ∅ DWrepresents the mean biomass concentration during the given time interval, *V* represents the total working volume, and *t* represents the cultivation time.

### Determination of the lipid content and fatty acid profile

After an appropriate amount of time for consuming nitrogen, strain JSC4 cells were harvested from the culture medium by centrifugation (7,000 rpm for 2 min). The cells were washed twice with deionized water, lyophilized, and weighed. The lipid composition was determined as fatty acid methyl esters (FAMEs) following direct transesterification of lipids according to the method described in Ho *et al*. [7]. FAMEs were analyzed by gas chromatography/mass spectrometry (GC/MS) on a GCMS-QP2010 Plus instrument (Shimadzu). Samples were injected onto a DB-23 capillary column (60 m, 0.25 mm internal diameter, 0.15-μm film thickness; Agilent Technologies, Palo Alto, CA, USA). Helium was used as the carrier gas at a flow rate of 2.3 mL min^−1^. The injector, ion source, and interface source temperatures were set at 230°C, 230°C, and 250°C, respectively. The oven temperature was initially set at 50°C for 1 min, increased from 50°C to 175°C at a rate of 25°C/min, increased from 175°C to 230°C at a rate of 4°C/min, and held at 230°C for 5 min. Purified FAMEs were identified based on retention time and the pattern of fragmentation by electron impact analysis. Supelco 37 Component FAME Mix (Sigma-Aldrich Co., St. Louis, MO, USA) was utilized as a quantitative standard, and pentadecanoic acid (Sigma-Aldrich Co.) was used as an internal standard.

### Sampling procedures for metabolic profiling

Cell sampling was performed according to our previously reported method [20], with minor modifications. Strain JSC4 cells (equivalent to 5 to 10 mg dry weight) were removed from cultivation vessels and added to a fourfold volume of prechilled (−30°C) quenching solution consisting of Modified Bold 3 N medium containing 32.5% methanol, then filtered using 1-μm pore-size Omnipore filter disks (Millipore, Billerica, MA, USA). After washing with 20 mM ammonium bicarbonate prechilled to 4°C, cells retained on the filters were immediately placed in 1 mL of prechilled (−30°C) methanol containing 12.4 μM piperazine-1,4-bis(2-ethanesulfonic acid) as an internal standard for mass analysis. Intracellular metabolites were extracted using cold 10:3:1 (*v*/*v*/*v*) methanol:chloroform:water combined with 30 min of bead-beater cell-breaking, as described previously [23]. Cells were suspended by vortexing and then 1 mL of the cell suspension was mixed with 100 μL of prechilled (4°C) water and 300 μL of chloroform. The cell suspension was shaken at 1,200 rpm in a model MBR-022UP incubator (TAITEC, Saitama, Japan) for 30 min at 4°C in the dark before centrifugation at 14,000 *g* for 5 min at 4°C. Next, 980 μL of the cell extract obtained as the supernatant was transferred to a clean tube. After adding 440 μL of water, the aqueous and organic layers were phase separated by centrifugation at 14,000 *g* for 5 min at 4°C. After filtration through a Millipore 5-kDa cut-off filter for the removal of solubilized proteins, the aqueous-layer extracts were evaporated under vacuum using a FreeZone 2.5 Plus freeze dry system (Labconco, Kansas City, MO, USA). Dried extracts were stored at −80°C until analysis by CE/MS.

### CE/MS metabolite analysis

Metabolites were determined according to the CE/MS method described by Hasunama *et al*. [20]. Dried metabolites were dissolved in 20 μL of Milli-Q water before analysis. The CE/MS experiments were performed using an Agilent G7100 CE system, an Agilent G6224AA LC/MSD time-of-flight (TOF) mass spectrometer, and an Agilent 1200 series isocratic HPLC pump equipped with a 1:100 splitter for delivery of the sheath liquid.

Agilent ChemStation software for CE and MassHunter software for the Agilent TOF-MS system were used for system control and data acquisition, respectively. The analytical conditions for anionic metabolite analyses were as described previously [20]. CE separations were performed using a fused silica capillary (1 m × 50 μm i.d.) filled with 50 mM ammonium acetate (pH 9) for anionic metabolite analyses. The CE polarity was such that the electrolyte vial (inlet) was at the anode, and the electrospray ionization (ESI) probe (outlet) was at the cathode. Samples were injected into the CE system at a pressure of 50 mbar for 30 s. The voltage applied to the CE capillary was set at 30 kV, with a ramp time of 0.3 min. For anionic metabolite analyses, the electrolyte solution was passed through the capillary using an air pump and was delivered at a pressure of 10 mbar between 0.4 and 30 min and 100 mbar between 30.1 and 49.5 min. The flow rate of the sheath liquid was set at 8 μL min^−1^. The ESI-MS analyses were conducted in either the positive or negative ion mode using a capillary voltage of −3.5 or 3.5 kV, respectively. The TOF-MS fragmenter, skimmer, and Oct RFV were set to 100, 65, and 750 V, respectively. The flow of heated drying nitrogen gas (300°C) was maintained at 10 L min^−1^. Mass data were acquired at a rate of 1 spectrum s^−1^ over the mass to charge ratio (*m*/*z*) range of 70 to 1,000.

### ^13^C-labeling

To analyze metabolic turnover in *Chlamydomonas* sp. JSC4, *in vivo*^13^C-labeling was performed using sodium ^13^C-bicarbonate (NaH^13^CO_3_) as a carbon source. Strain JSC4 was precultivated in Modified Bold 3 N medium or Modified Bold 1 N medium at a light intensity of 30 μmol m^−2^ s^−1^ or 300 μmol m^−2^ s^−1^, respectively, for approximately 2 to 3 days and then resuspended in Modified Bold 3 N or Modified Bold 0 N medium containing 25 mM NaH^13^CO_3_. After labeling for 1 to 30 min, 5 to 10 mg of strain JSC4 cells were collected by filtration and processed as described in the previous section. Extracted intracellular metabolites were then analyzed using CE/MS. Mass spectral peaks of biological origin were identified manually by searching for mass shifts between ^12^C- and ^13^C-mass spectra. The ^13^C fraction of each metabolite and the metabolic turnover rate were determined as described previously [40].

## Additional file

Additional file 1: Table S1. Comparison of key metabolite pools. Comparison of key metabolite pools in *Chlamydomonas* sp. JSC4 under cells cultivated under low light intensity of 30 μmol m^−2^ s^−1^, nitrogen-rich conditions; high light intensity of 300 μmol m^−2^ s^−1^, nitrogen-rich conditions; or high light intensity of 300 μmol m^−2^ s^−1^, nitrogen-free conditions. Values are the mean ± standard deviation of three replicated experiments.

## Abbreviations

3-PG: 3-phosphoglycerate
AceCoA: Acetyl-CoA
CE/MS: Capillary electrophoresis coupled with mass spectrometry
DCW: Dry cell weight
ESI: Electrospray ionization
F6P: Fructose-6-phosphate
FAMEs: Fatty acid methyl esters
G1P: Glucose-1-phosphate
G3P: Glycerol-3-phosphate
G6P: Glucose-6-phosphate
GC/MS: Gas chromatography/mass spectrometry
OD: Optical density
PBR: Photobioreactor
PEP: Phosphoenolpyruvate
Pyr: Pyruvate
TAG: Triacylglycerol
TOF: Time-of-flight
