# Supplementary material for: Dynamic metabolic profiling of the marine microalga Chlamydomonas sp. JSC4 and enhancing its oil production by optimizing light intensity
Source: Biotechnol Biofuels. 2015 Mar 18;8:48. doi: 10.1186/s13068-015-0226-y (PMC4369889; doi:10.1186/s13068-015-0226-y)
Supplement: Additional file 1: Table S1. — Comparison of key metabolite pools. Comparison of key metabolite pools in Chlamydomonas sp. JSC4 under cells cultivated under low light intensity of 30 μmol m−2 s−1, nitrogen-rich conditions; high light intensity of 300 μmol m−2 s−1, nitrogen-rich conditions; or high light intensity of 300 μmol m−2 s−1, nitrogen-free conditions. Values are the mean ± standard deviation of three replicated experiments. [file 13068_2015_226_MOESM1_ESM.docx]

**Supplemental Table 1** Comparison of key metabolite pools in *Chlamydomonas* sp. JSC4 under cells cultivated under low light intensity of 30 μmol m^−2^s^−1^, nitrogen-rich conditions; high light intensity of 300 μmol m^−2^s^−1^, nitrogen-rich conditions; or high light intensity of 300 μmol m^−2^s^−1^, nitrogen-free conditions. Values are the mean ± standard deviation of three replicated experiments. Abbreviations: AceCoA, acetyl-CoA; F6P, fructose-6-phosphate; G1P, glucose-1-phosphate; G6P, glucose-6-phosphate; PEP, phosphoenolpyruvate; 3-PG, 3-phosphoglycerate; Pyr, pyruvate.

| Metabolites | Pool size (nmol mg^-1^) | | |
| --- | --- | --- | --- |
|  | 30 μmol m^-2^s^-1^_N-rich | 300 μmol m^-2^s^-1^_N-rich | 300 μmol m^-2^s^-1^_N-free |
| F6P | 0.209 ± 0.094 | 0.174 ± 0.003 | 0.108 ± 0.005 |
| G6P | 0.320 ± 0.066 | 0.224 ± 0.015 | 0.170 ± 0.001 |
| G1P | 0.036 ± 0.003 | 0.041 ± 0.004 | 0.012 ± 0.004 |
| 3-PG | 0.995 ± 0.349 | 1.181 ± 0.085 | 1.364 ± 0.371 |
| PEP | 0.110 ± 0.048 | 0.050 ± 0.028 | 0.071 ± 0.024 |
| Pyr | 0.321 ± 0.032 | 0.591 ± 0.009 | 0.683 ± 0.224 |
| AceCoA | 0.014 ± 0.002 | 0.028 ± 0.006 | 0.014 ± 0.003 |
